# Influence of age on the diagnostic accuracy of soluble biomarkers for tuberculous pleural effusion: a post hoc analysis

**DOI:** 10.1186/s12890-020-01219-2

**Published:** 2020-06-22

**Authors:** Chun-Guo Jiang, Wen Wang, Qiong Zhou, Xiu-Zhi Wu, Xiao-Juan Wang, Zhen Wang, Kan Zhai, Huan-Zhong Shi

**Affiliations:** 1 Department of Respiratory and Critical Care Medicine, Beijing Chaoyang Hospital, Capital Medical University, 8 Gongti Nanlu, Chaoyang District, Beijing, 100020 China; 2 Department of Respiratory and Critical Care Medicine, Union Hospital, Tongji Medical College, Huazhong University of Science and Technology, Wuhan, China

**Keywords:** Adenosine deaminase, Interferon-gamma, Interleukin 27, Tuberculous pleural effusion

## Abstract

**Background:**

Accurately diagnosing pleural effusion is a frequent and significant problem in clinical practice. Combining pleural biomarkers with patients’ age may be a valuable method for diagnosing TPE. We sought to evaluate the influence of age on diagnostic values of pleural adenosine deaminase (ADA), interferon-gamma (IFN-γ), and interleukin 27 (IL-27) for tuberculous pleural effusion (TPE).

**Methods:**

Two hundred seventy-four consecutive adult patients with pleural effusion were selected from Beijing and Wuhan between January 1, 2014 and June 30, 2015, and their pleural fluid concentrations of ADA, IFN-γ, and IL-27 were tested. Biomarker performance was analyzed by standard receiver operating characteristic (ROC) curves according to different ages.

**Results:**

Data from the Beijing cohort showed that ADA, IFN-γ, and IL-27 could all accurately diagnose TPE in young patients (≤ 40 years of age). With a cutoff of 21.4 U/L, the area under the curve (AUC), sensitivity, specificity, positive predictive value (PPV), and negative predictive value (NPV) of ADA for diagnosing TPE were 1.000 (95% confidence interval: 0.884–1.000), 100.0, 100.0%, 100.0, and 100.0, respectively. In older patients (> 40 years of age), IL-27 and IFN-γ were excellent biomarkers for discriminating TPE versus non-TPE cases. With a cutoff of 591.4 ng/L, the AUC, sensitivity, specificity, PPV, and NPV of IL-27 for diagnosing TPE were 0.976 (95% confidence interval: 0.932–0.995), 96.3, 99.0%, 96.3, and 99.0, respectively. Similar diagnostic accuracy among the three pleural biomarkers was validated in the Wuhan cohort.

**Conclusions:**

Among young patients, ADA is reliable for diagnosing TPE. Conversely, in older patients, IL-27 and IFN-γ are excellent biomarkers to differentiate TPE versus non-TPE cases.

## Background

Tuberculosis is a serious public health problem globally but especially in developing countries. Tuberculous pleural effusion (TPE) ranks among the most regular forms of extrapulmonary tuberculosis [1]. The definitive diagnosis of TPE relies upon the presence of *Mycobacterium tuberculosis* in the sputum, pleural effusion, or pleural biopsy specimens [2]. To achieve the gold standard, conventional diagnostic tests include microscopic examination and/or culturing of pleural fluid, sputum, or pleural tissue for acid-fast bacilli or the histopathological demonstration of caseating granulomas in the pleura along with acid-fast bacilli. These tests have recognized limitations in clinical practice, such as low sensitivity, lengthy delay, or invasiveness [3, 4]; as a result, multiple biomarkers in pleural effusion have been investigated, including adenosine deaminase (ADA) and interferon-gamma (IFN-γ) [5].

Differences in the diagnostic value of the same biomarker in the literature is associated with a variety of factors. One such factor is age, which has been reported to be correlated with pleural ADA levels and affects the diagnostic accuracy of TPE [6–11]. One possible explanation is that elderly people suffer a degradation of immune status with aging, believed to result from a functional decrease in lymphocyte and macrophage levels [10], and this may affect the levels of pleural biomarkers. However, it is still less clear as to whether combining pleural biomarkers with patients’ age could be a valuable method for estimating the probability of TPE.

We conducted two prospective studies and one meta-analysis, finding that the diagnostic value of interleukin 27 (IL-27) is comparable to that of IFN-γ and more accurate than that of ADA [12]. Through a post-hoc analysis of the two independent prospective blind studies, we focused on the influence of age, as an independent covariate, on the diagnostic accuracy of ADA, IFN-γ, and IL-27 in pleural fluid to differentiate between TPE and non-TPE cases.

## Methods

### Study populations, diagnostic criteria, sample collection, and determination

As previously described [12], our cohort included 154 consecutively enrolled adult patients (≥18 years old) from Beijing Chaoyang Hospital (Beijing cohort) and 120 consecutively enrolled patients from Union Hospital, Tongji Medical College (Wuhan cohort). Enrollment occurred between January 1, 2014 and June 30, 2015. Approvals were granted for the conduct of this study by the appropriate ethics committees (No. 2012-S068, 2014-ke-63). Based on the established criteria, cases were classified as either TPE, malignant pleural effusion (MPE), parapneumonic pleural effusion (PPE), and miscellaneous pleural effusion [12]. Pleural fluid was obtained by the first successful thoracentesis before treatment initiation and was measured using colorimetric method kits for ADA activity and enzyme-linked immunosorbent assay kits for IFN-γ and IL-27. The technicians performing the assays were unaware of the nature of the samples, and the statisticians broke the code to analyze the database.

### Statistical analysis

Data are presented as means ± standard errors of the means or medians with 25th and 75th percentiles, depending on normal distribution by the Kolmogorov–Smirnov test. For group comparisons, the Student’s *t* test, Mann–Whitney *U* test, one-way analysis of variance (ANOVA), or Kruskal–Wallis ANOVA on ranks were used. Receiver operating characteristic (ROC) curves were drawn to evaluate the diagnostic performance of the three biomarkers in pleural effusion. Areas under the curves (AUCs) were computed and compared using the Hanley and McNeil procedure [13], including sensitivity, specificity, positive and negative likelihood ratios (PLR and NLR), and positive and negative predictive values (PPV and NPV) [14]. The parameters of diagnostic accuracy are shown together with their 95% confidence intervals (CIs). ROCs of the same tests in independent populations were compared by using the method proposed by Gönen M [15].

The optimal cutoff values of ADA, IFN-γ, and IL-27 obtained in the Beijing cohort were 21.4 U/L, 116.1 ng/L, and 591.4 ng/L, respectively, and these were verified in the Wuhan cohort [12]. In the present study, to determine the diagnostic accuracy of each biomarker in different ages separately, cases complying with the cutoff values were considered as positive. The data were analyzed using the Statistical Package for the Social Sciences (IBM Corp., Armonk, NY, USA) and MedCalc software (MedCalc Software, Ostend, Belgium) and *p* < 0.05 was considered statistically significant.

## Results

### Clinical characteristics

The baseline characteristics of the 274 patients recruited in both cohorts are summarized in Table 1. According to previous literatures [16, 17] and our preliminary statistics, we selected 40 years old as the cut point for two age groups: young patients (≥18 years to 40 years of age) and older patients (> 40 years of age). In both cohorts, as compared with older patients, the prevalence of TPE was relatively high (80% in Beijing and 70% in Wuhan) in young patients. There were no differences in demographic characteristics between the Beijing and Wuhan cohorts.

**Table 1:** Demographic characteristics^a^

		Beijing cohort				Wuhan cohort			
	Variable	TPE	MPE	PPE	Miscellaneous	TPE	MPE	PPE	Miscellaneous
≤40	n	24	3	1	2	14	3	2	1
	Sex, male, %	62.5	0	100	50	85.7	100	100	0
	Age, years	26.7 ± 1.2	37.7 ± 1.9	25	28.5 ± 3.5	29.2 ± 2.1	36.7 ± 2.8	35.5 ± 0.5	28
> 40	n	27	58	27	12	30	44	17	9
	Sex, male, %	66.7	60.3	85.2	91.7	70	52.3	76.5	55.6
	Age, years	57.9 ± 2.5	64.5 ± 1.4	62.9 ± 2.0	67.3 ± 3.2	57.4 ± 2.4	60.0 ± 1.3	65.3 ± 3.3	57.6 ± 3.6

^a^Data are presented as means ± SEMs. *MPE* malignant pleural effusion; *PPE* parapneumonic pleural effusion; *TPE* tuberculous pleural effusion

### ADA, IFN-γ, and IL-27 concentrations

Regardless of age, the concentrations of ADA, IFN-γ, and IL-27 in TPE patients were all significantly higher than those in non-TPE patients in each cohort and in the combined population (all *p* < 0.001) (Table 2), and no differences were found among MPE, PPE, or miscellaneous effusions (all *p* > 0.05). We also noted that, either among all patients with pleural effusion or TPE patients, there was no difference in the concentrations of ADA, IFN-γ, or IL-27 between age groups in both cohorts (all p > 0.05).

**Table 2:** Concentrations of ADA, IFN-γ, and IL-27 in pleural fluid according to study cohort and age*

		Beijing cohort (*n* = 154)				Wuhan cohort (*n* = 120)				Total (*n* = 274)			
	Variable	TPE	MPE	PPE	Miscellaneous	TPE	MPE	PPE	Miscellaneous	TPE	MPE	PPE	Miscellaneous
≤40	ADA, U/L	55.6 ± 5.3†	8.47 ± 2.4	2.6	8.5 ± 2.4	62.0 ± 12.3†	9.0 ± 1.4	14.1 ± 2.4	21.0	58.0 ± 5.5†	8.7 ± 1.3	10.2 ± 4.1	9.8 ± 3.2
	IFN-γ, ng/L	3407.1 ± 736.2‡	44.7 ± 8.7	42.9	44.7 ± 8.7	2297.5 ± 543.0‡	16.4 ± 1.4	14.8 ± 0.8	104.7	2998.2 ± 508.6‡	30.5 ± 7.4	24.2 ± 9.4	49.6 ± 9.7
	IL-27, ng/L	875.8 ± 51.3†	279.0 ± 24.9	198.2	279.0 ± 24.9	853.8 ± 49.8†	240.4 ± 37.7	452.7 ± 138.4	240.4	867.7 ± 36.9†	259.7 ± 22.0	367.9 ± 116.5	381.9 ± 106.7
>40	ADA, U/L	43.5 ± 4.9†	16.0 ± 1.4	14.9 ± 1.6	16.1 ± 2.2	41.8 ± 4.0†	13.6 ± 1.3	14.7 ± 1.3	18.3 ± 2.2	42.7 ± 4.4†	14.8 ± 1.3	14.8 ± 1.4	17.2 ± 2.2
	IFN-γ, ng/L	2176.4 ± 641.9‡	35.7 ± 3.3	59.8 ± 17.4	102.0 ± 54.6	2508.5 ± 660.5‡	41.3 ± 7.3	67.3 ± 10.5	111.9 ± 63.5	2342.2 ± 651.2‡	38.5 ± 5.2	63.6 ± 14.0	107.0 ± 59.1
	IL-27, ng/L	907.7 ± 49.1†	299.3 ± 13.8	273.1 ± 16.5	251.7 ± 14.1	900.0 ± 52.9†	292.0 ± 13.5	289.6 ± 18.1	253.6 ± 20.5	903.9 ± 51.0†	295.7 ± 13.6	281.3 ± 17.3	252.7 ± 17.3

*Data are presented as mean ± SEM or median (25th - 75th centile). †p < 0.001 compared with each non-TPE group using analysis of variance (ANOVA) followed by Bonferroni’s test; ‡p < 0.001 compared with the corresponding serum using paired *t* test. ADA, adenosine deaminase; IFN, interferon; IL, interleukin; MPE, malignant pleural effusion; PPE, parapneumonic pleural effusion; TPE, tuberculous pleural effusion

### Diagnostic accuracy of ADA, IFN-γ, and IL-27 in the Beijing cohort

**Fig. 1:**
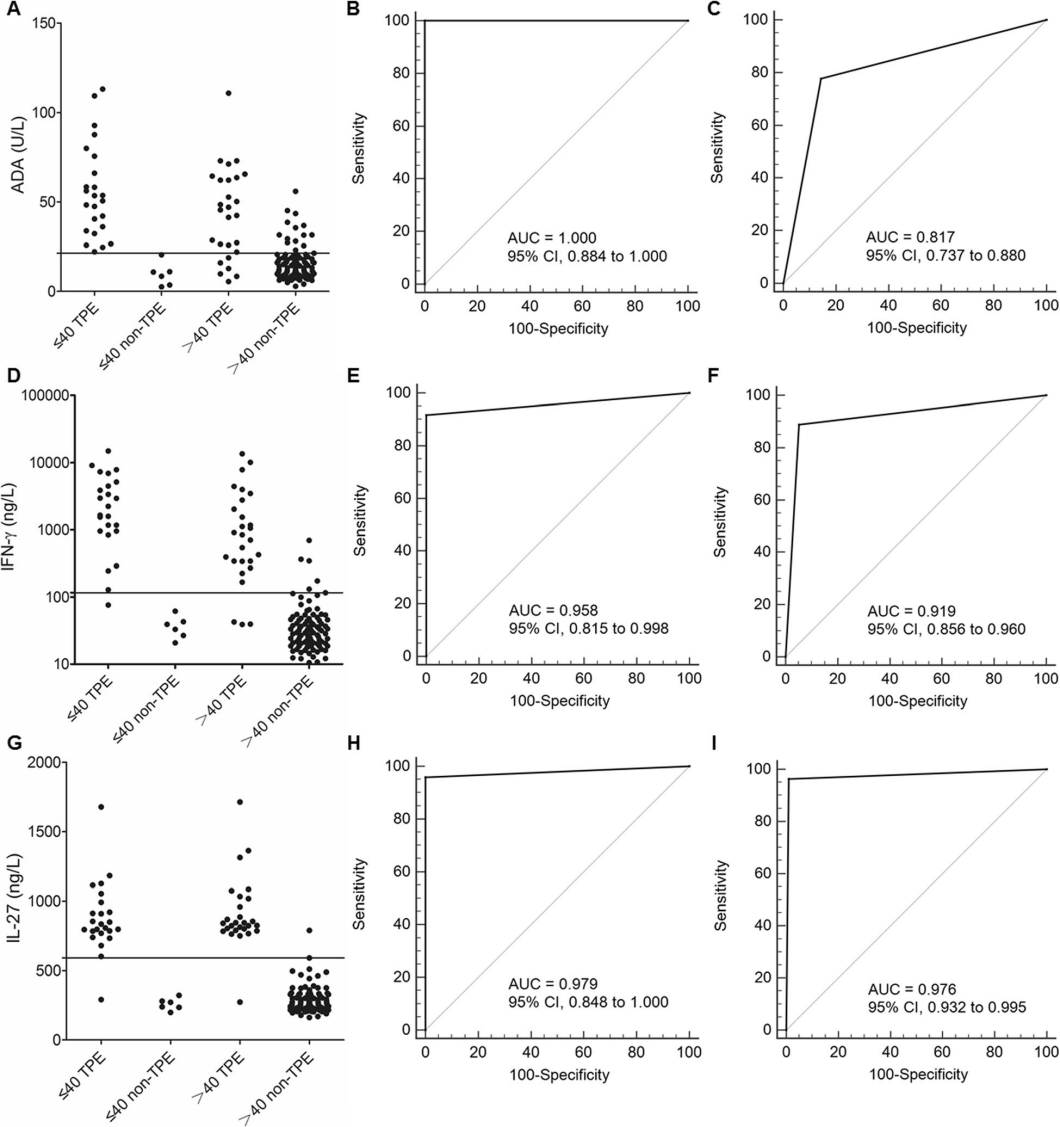
Diagnostic accuracy of ADA, IFN-γ, and IL-27 for TPE according to different ages in the Beijing Cohort. A cutoff point of 21.4 U/L for ADA to discriminate TPE from non-TPE (**a**). ROC curves show the diagnostic value of ADA in young patients (**b**) and in older patients (**c**). A cutoff point of 116.1 ng/L for IFN-γ to discriminate TPE from non-TPE (**d**). ROC curves show the diagnostic value of pleural IFN-γ in young patients (**e**) and in older patients (**f**). A cutoff point of 591.4 ng/L for IL-27 to discriminate TPE from non-TPE (**g**). ROC curves show the diagnostic value of pleural IL-27 in young patients (**h**) and in older patients (**i**). ADA, adenosine deaminase; IFN, interferon; IL, interleukin; ROC, receiver operating characteristic; TPE, tuberculous pleural effusion

The capacity of ADA, IFN-γ, and IL-27 to diagnose TPE was assessed using ROC curve analysis in the Beijing cohort (Table 3 and Fig. 1). In young patients, ADA, IFN-γ, and IL-27 all accurately discriminated TPE and non-TPE (*p* < 0.001). It’s worth noting that, with a cutoff of 21.4 U/L, the AUC of ADA in discriminating TPE from non-TPE was 1.000 (95% CI: 0.884–1.000; p < 0.001), while the sensitivity, specificity, NLR, PPV, and NPV of ADA were 100.0, 100.0%, 0, 100.0, and 100.0, respectively. Meanwhile, the AUCs of IFN-γ and IL-27 were 0.958 (95% CI: 0.815–0.998) and 0.979 (95% CI: 0.848–1.000), respectively. Z-statistical analysis further revealed that the AUC (95% CI) of ADA was not significantly different from that of IFN-γ [0.042 (− 0.028 to 0.112); z = 1.167; *p* = 0.243] or IL-27 [0.021 (− 0.028 to 0.070); z = 0.829; *p* = 0.407].

**Table 3:** Diagnostic performance of pleural IL-27, ADA, and IFN-γ in differentiating between patients with tuberculous pleural effusion (TPE) and those with non-TPEs in the Beijing cohort (n = 154)

Age	Variable	Cut-off value	AUC (95% CI)	Sensitivity (%)	Specificity (%)	PLR	NLR	PPV	NPV
≤40	ADA	21.4 U/L	1.000 (0.884 to 1.000)	100.0 (85.8 to 100.0)	100.0 (54.1 to 100.0)	–	0	100.0	100.0
	IFN-γ	116.1 ng/L	0.958 (0.815 to 0.998)	91.7 (73.0 to 99.0)	100.0 (54.1 to 100.0)	–	0.1 (0 to 0.3)	100.0	75.0 (44.3 to 91.9)
	IL-27	591.4 ng/L	0.979 (0.848 to 1.000)	95.8 (78.9 to 99.9)	100.0 (54.1 to 100.0)	–	0 (0 to 0.3)	100.0	85.7 (46.8 to 97.6)
>40	ADA	21.4 U/L	0.817 (0.737 to 0.880)	77.8 (57.7 to 91.4)	85.6 (77.0 to 91.9)	5.4 (3.2 to 9.1)	0.3 (0.1 to 0.5)	60.0 (47.0 to 71.7)	93.3 (87.2 to 96.6)
	IFN-γ	116.1 ng/L	0.919* (0.856 to 0.960)	88.9 (70.8 to 97.6)	94.9 (88.4 to 98.3)	17.2 (7.3 to 40.9)	0.1 (0 to 0.3)	82.8 (66.9 to 91.9)	96.8 (91.3 to 98.9)
	IL-27	591.4 ng/L	0.976† (0.932 to 0.995)	96.3 (81.0 to 99.9)	99.0 (94.4 to 100.0)	93.4 (13.3 to 657.4)	0 (0 to 0.3)	96.3 (78.7 to 99.5)	99.0 (93.3 to 99.8)

*p < 0.05, †p < 0.05, compared with ADA using the z statistic, respectively. *ADA* adenosine deaminase; *AUC* area under the curve; *IFN* interferon; *IL* interleukin; *NLR* negative likelihood ratio; *NPV* negative predictive value; *PLR* positive likelihood ratio; *PPV* positive predictive value

In the subgroup of older patients, with a cutoff of 591.4 ng/L, IL-27 presented the largest AUC (95% CI) [0.976 (0.932–0.995); *p* < 0.001] to differentiate TPE and non-TPE (Table 3 and Fig. 1). The sensitivity, specificity, PLR, NLR, PPV, and NPV of IL-27 were 96.3, 99.0%, 93.4, 0, 96.3, and 99.0, respectively. The AUCs (95% CIs) of ADA and IFN-γ were 0.817 (0.737–0.880) and 0.919 (0.856–0.960), respectively. The AUC (95% CI) of IL-27 was much higher than that of ADA [0.160 (0.057–0.262); z = 3.048; *p* = 0.002] but not significantly higher than that of IFN-γ [0.058 (− 0.027 to 0.142); z = 1.339; *p* = 0.181].

The AUC of ADA in young patients was significantly higher than that in older patients [0.183 (0.083–0.283); z = 3.594; *p* < 0.001]; however, there were no significant differences between young patients and older patients in terms of IFN-γ [0.040 (− 0.062 to 0.141); z = 0.765; *p* = 0.444] or IL-27 [0.003 (− 0.063 to 0.068); z = 0.085; *p* = 0.932].

### Diagnostic accuracy of ADA, IFN-γ, and IL-27 in the Wuhan cohort

**Fig. 2:**
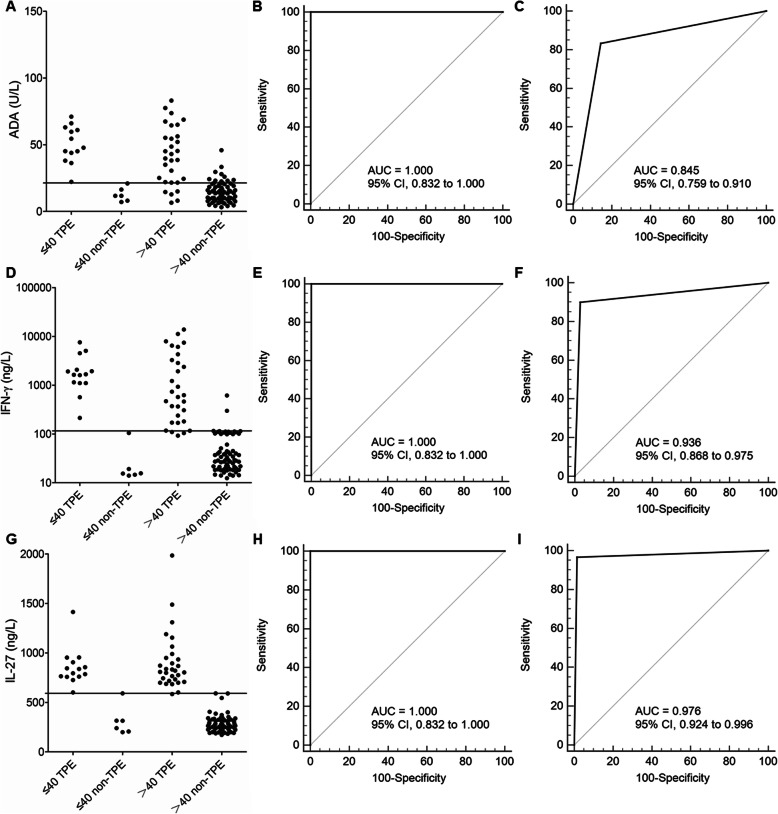
Diagnostic accuracy of ADA, IFN-γ and IL-27 for TPE according to different ages in the Wuhan Cohort. A cutoff point of 21.4 U/L for ADA to discriminate TPE from non-TPE (**a**). ROC curves show the diagnostic value of ADA in young patients (**b**) and in older patients (**c**). A cutoff point of 116.1 ng/L for IFN-γ to discriminate TPE from non-TPE (**d**). ROC curves show the diagnostic value of pleural IFN-γ in young patients (**e**) and in older patients (**f**). A cutoff point of 591.4 ng/L for IL-27 to discriminate TPE from non-TPE (**g**). ROC curves show the diagnostic value of pleural IL-27 in young patients (**h**) and in older patients (**i**). ADA, adenosine deaminase; IFN, interferon; IL, interleukin; ROC, receiver operating characteristic; TPE, tuberculous pleural effusion

For further validation of the diagnostic accuracy of ADA, IFN-γ, and IL-27 obtained from the Beijing cohort, we performed another prospective blinded study among the Wuhan cohort – a large urban city with high prevalence of tuberculosis (Table 4 and Fig. 2). In young patients, ADA, IFN-γ, and IL-27 were all perfect for diagnosing TPE (*p* < 0.001), presenting AUCs (95% CIs) of 1.000 (0.832–1.000), 1.000 (0.832–1.000), and 1.000 (0.832–1.000), respectively, without significant differences. The AUCs in the Wuhan cohort were similar to the Beijing cohort (ADA: T = 0, *p* = 1.000; IFN-γ: T = 0.014, *p* = 0.906; IL-27: T = 0.700, *p* = 0.403).

**Table 4:** Diagnostic performance of pleural ADA, IFN-γ, and IL-27 in differentiating between patients with tuberculous pleural effusion (TPE) and those with non-TPEs in the Wuhan cohort (n = 120)

	Variable	Cut-off value	AUC (95% CI)	Sensitivity (%)	Specificity (%)	PLR	NLR	PPV	NPV
≤40	ADA	21.4 U/L p < 0.001	1.000 (0.832 to 1.000)	100.0 (76.8 to 100.0)	100.0 (54.1 to 100.0)	–	0	100.0	100.0
	IFN-γ	116.1 ng/L p < 0.001	1.000 (0.832 to 1.000)	100.0 (76.8 to 100.0)	100.0 (54.1 to 100.0)	–	0	100.0	100.0
	IL-27	591.4 ng/L p < 0.001	1.000 (0.832 to 1.000)	100.0 (76.8 to 100.0)	100.0 (54.1 to 100.0)	–	0	100.0	100.0
>40	ADA	21.4 U/L p < 0.001	0.845 (0.759 to 0.910)	83.3 (65.3 to 94.4)	85.7 (75.3 to 92.9)	5.8 (3.2 to 10.6)	0.2 (0.1 to 0.4)	71.4 (57.9 to 81.9)	92.3 (84.3 to 96.4)
	IFN-γ	116.1 ng/L p < 0.001	0.936* (0.868 to 0.975)	90.0 (73.5 to 97.9)	97.1 (90.1 to 99.7)	31.5 (8.0 to 124.1)	0.1 (0 to 0.3)	93.1 (77.4 to 98.2)	95.8 (88.6 to 98.5)
	IL-27	591.4 ng/L p < 0.001	0.976† (0.924 to 0.996)	96.7 (82.8 to 99.9)	98.6 (92.3 to 100.0)	67.7 (9.7 to 474.2)	0 (0 to 0.2)	96.7 (80.5 to 99.5)	98.6 (90.9 to 99.8)

*p < 0.05, †p < 0.05, compared with ADA using the z statistic, respectively. *ADA* adenosine deaminase; *AUC* area under the curve; *IFN* interferon; *IL* interleukin; *NLR* negative likelihood ratio; *NPV* negative predictive value; *PLR* positive likelihood ratio; *PPV* positive predictive value

In older patients, excellent discrimination between TPE and non-TPE for IL-27 was observed [AUC: 0.976 (95% CI: 0.924–0.996); *p* < 0.001] (Table 4 and Fig. 2). The AUC of IL-27 was not higher than that of IFN-γ [0.041 (− 0.037 to 0.118_; z = 1.024; *p* = 0.306] but was significantly higher than that of ADA [0.131 (0.033–0.229); z = 2.623; *p* = 0.009]. Additionally, the AUC (95% CI) of ADA was lower than that of IFN-γ [0.091 (0.009–0.172); z = 2.168; *p* = 0.030]. The AUCs in the Wuhan cohort were similar to the Beijing cohort (ADA: T = 0.165, *p* = 0.685; IFN-γ: T = 0.162, *p* = 0.738; IL-27: T = 0, *p* = 1.000).

The AUC (95% CI) of ADA was significantly lower among older patients than young patients [0.155 (0.064–0.246]; z = 3.338; p < 0.001] without significant differences for IFN-γ [0.064 (− 0.003 to 0.131); z = 1.886; *p* = 0.059] or IL-27 [0.024 (− 0.017 to 0.065); z = 1.148; *p* = 0.251].

Thus, we confirmed that the diagnostic accuracies of the above three pleural biomarkers were similar in the two cohorts.

## Discussion

Diagnosing pleural effusion remains a challenge in clinical practice. Considering different etiologies, tuberculosis is the most common cause of pleural effusion in developing countries [18, 19]. Unfortunately, conventional methods of microscopic examination, culture, or histopathological demonstration for diagnosing TPE have shown low sensitivity or are expensive and invasive [2, 20]. A number of pleural soluble biomarkers have been evaluated [5, 12, 19], yet a problem of insufficient overall diagnostic accuracy has consistently been encountered.

Several studies have introduced predictive models based on clinical parameters and the chemistry profile in pleural fluid to facilitate differential diagnosis between TPE and non-TPE [21], but few studies have looked into the factors affecting pleural biomarkers, especially in relation to age [6–11]. The majority of published studies are retrospective single-center ones and lack validation. In this post-hoc analysis of two independent prospective blinded studies, we evaluated the diagnostic values of ADA, IFN-γ, and IL-27 in pleural fluid for TPE according to different ages. Our results showed that the levels of these biomarkers were not different between young patients and older patients. In young patients, ADA, IFN-γ, and IL-27 accurately diagnosed TPE, while in older patients, IL-27 and IFN-γ appeared as excellent options to discriminate TPE from non-TPE. Age affected the diagnostic accuracy of pleural ADA for TPE, with a trend towards a reduction in older patients. Of note, this is, to the best of our knowledge, the first study to examine factors affecting pleural IL-27.

Several studies have demonstrated a negative correlation between pleural fluid ADA activity and age in the entire cohort and/or in a subgroup of patients with TPE [6–11], as age-associated immune decline is likely to affect ADA level. However, we did not find any difference between young patients and older patients regarding the concentrations of ADA, IFN-γ, and IL-27. This discrepancy may be partly related to the study inclusion criteria, the number of participants, and variations in age groups. Thus, the true role of age-associated changes in pleural biomarker levels and the precise mechanisms remains an important area for future inquiry/investigation.

Several previous studies have reported relatively high diagnostic accuracy for pleural ADA, IFN-γ, and IL-27 for TPE [12, 22, 23]. One published study suggested that, in a region with a high prevalence of tuberculosis, the routine pleural fluid measurement of ADA concentration among young patients displays a reliable level of diagnostic accuracy [24]. Our research confirmed these earlier findings and further supports the use of ADA, IFN-γ, and IL-27 as excellent diagnostic biomarkers to discriminate TPE from non-TPE in young patients with high sensitivity and specificity values of 100.0 and 100.0%, respectively. Each of these three biomarkers can be used as a rule-in test for TPE when they surpass their threshold values. TPE patients had a lower mean age, and tuberculosis was reported as the most common cause of pleural effusion in patients under 40 years of age in areas with a high incidence of tuberculosis [16], so the higher diagnostic accuracy of these biomarkers may partly be due to the increased prevalence of TPE. Some authors have also reported high ADA levels among patients with other causes of pleural effusion (e.g., pneumonia, empyema, lymphomas, adenocarcinomas, and systemic lupus erythematosus), but all of our young patients with these conditions showed pleural ADA concentrations below our diagnostic threshold. Most TPE cases could be diagnosed by medical thoracoscopy [25]; however, not everyone is a candidate for or willing to undergo thoracoscopy for its invasiveness. Since the detection of ADA is simpler and cheaper, the present study recommends it as the first choice for the diagnosis of TPE in young patients [26], may help to avoid further invasive biopsies in some cases.

IL-27, a member of the IL-12 cytokine family produced by activated antigen-presenting cells, has been reported to control the development of regulatory T cells and IL-17–producing CD4+ T cells [27]. Our previous study showed that the diagnostic performance of IL-27 was comparable to that of IFN-γ and more accurate than that of ADA in the diagnosis of TPE in the total patient population [12]. In the present study, we once again observed that, in both cohorts, IL-27 and IFN-γ constitute excellent biomarkers to diagnose TPE in older patients and their diagnostic values are not affected by patient age. As the predictive value of pleural fluid ADA level for TPE decreases with increasing age, physicians should be cautious when interpreting the pleural ADA level to diagnose TPE if the patient is older. Some studies have suggested that the diagnostic cutoff value of pleural ADA activity for TPE requires adjustment according to different age groups [6, 8]. Although IFN-γ has an advantage in diagnosing TPE, its daily clinical use is limited due to the high cost and lack of acceptable cutoff value [2, 19]. Due to the excellent diagnostic accuracy of pleural IL-27 for TPE in this study, such represents a potential option in routine clinical practice in older patients.

Based on the findings of our study, in regions of high tuberculosis prevalence, we hypothesize that combining pleural biomarkers with patients’ age should be a simple method for estimating the probability of TPE with high sensitivity and specificity. If patients are younger than 40 years of age, we recommend testing for pleural ADA, while, if patients are older than 40 years of age, then IL-27 should be measured. Due to the high diagnostic accuracy of pleural biomarkers according to different ages for TPE, one might consider skipping unnecessary invasive diagnostic procedures and initiating antituberculous therapy in certain groups of patients, and the study holds a certain meaning for health economics, especially in developing countries.

Several limitations of this study should not be ignored. First, although it provided data from two separate prospective cohorts containing more than 250 patients, the total number of cases was still small, especially in the young non-TPE group, as other causes of pleural effusion in young patients were relatively rare from the epidemiological perspective, and the small study sample may be affected by selection bias. Second, the prevalence of tuberculosis, time of pleural fluid collection, standardization of the detection method, or cutoff point may all influence the study results, and a subgroup analysis controlling for confounders was not conducted. Third, our simple flowchart, consisting of age and pleural biomarkers, is useful and accurate for TPE diagnosis. However, it is just a first step and should not be considered as an alternative to biopsy and culture. More invasive methods or other means should potentially follow to establish a definitive diagnosis of TPE. In addition, further studies are required to confirm the mechanism associated with the biomarkers in pleural fluids.

## Conclusions

In conclusion, our study reports the use of pleural biomarkers according to different ages may improve the diagnostic accuracy for TPE. In high tuberculosis prevalence settings, in young patients, ADA, IFN-γ, and IL-27 could all accurately diagnosis TPE. In older patients, IL-27 and IFN-γ are excellent biomarkers to discriminate TPE from non-TPE. However, additional studies are needed to provide more convincing data to establish the definite diagnosis of pleural effusion for clinical practice, especially before the results of biopsy or culture become available.

## Data Availability

The datasets used and/or analysed during the current study are available from the corresponding author on reasonable request.

## Abbreviations

ADA: Adenosine deaminase
ANOVA: Analysis of variance
AUC: Area under the curve
CI: Confidence interval
IFN: Interferon
IL: Interleukin
MPE: Malignant pleural effusion
NLR: Negative likelihood ratio
NPV: Negative predictive value
PLR: Positive likelihood ratio
PPE: Parapneumonic pleural effusion
PPV: Positive predictive value
ROC: Receiver operating characteristic
TPE: Tuberculous pleural effusion
